# qBase relative quantification framework and software for management and automated analysis of real-time quantitative PCR data

**DOI:** 10.1186/gb-2007-8-2-r19

**Published:** 2007-02-09

**Authors:** Jan Hellemans, Geert Mortier, Anne De Paepe, Frank Speleman, Jo Vandesompele

**Affiliations:** 1 Center for Medical Genetics, Ghent University Hospital, De Pintelaan, B-9000 Ghent, Belgium

## Abstract

Although quantitative PCR (qPCR) is becoming the method of choice for expression profiling of selected genes, accurate and straightforward processing of the raw measurements remains a major hurdle. Here we outline advanced and universally applicable models for relative quantification and inter-run calibration with proper error propagation along the entire calculation track. These models and algorithms are implemented in qBase, a free program for the management and automated analysis of qPCR data.

## Abstract 2

qBase, a free program for the management and automated analysis of qPCR data, is described

## Background

Since its introduction more than 10 years ago [1], quantitative PCR (qPCR) has become the standard method for quantification of nucleic acid sequences. The ease of use and high sensitivity, specificity and accuracy has resulted in a rapidly expanding number of applications with increasing throughput of samples to be analyzed. The software programs provided along with the various qPCR instruments allow for straightforward extraction of quantification cycle values from the recorded fluorescence measurements, and at best, interpolation of unknown quantities using a standard curve of serially diluted known quantities. However, these programs usually do not provide an adequate solution for the processing of these raw data (coming from one or multiple runs) into meaningful results, such as normalized and calibrated relative quantities. Furthermore, the currently available tools all have one or more of the following intrinsic limitations: dedicated for one instrument, cumbersome data import, a limited number of samples and genes can be processed, forced number of replicates, normalization using only one reference gene, lack of data quality controls (for example, replicate variability, negative controls, reference gene expression stability), inability to calibrate multiple runs, limited result visualization options, lack of experimental archive, and closed software architecture.

To address the shortcomings of the available software tools and quantification strategies, we modified the classic delta-delta-Ct method to take multiple reference genes and gene specific amplification efficiencies into account, as well as the errors on all measured parameters along the entire calculation track. On top of that, we developed an inter-run calibration algorithm to correct for (often underestimated) run-to-run differences.

Our advanced models and algorithms are implemented in qBase, a flexible and open source program for qPCR data management and analysis. Four basic principles were followed during development of the program: the use of correct models and formulas for quantification and error propagation, inclusion of data quality control where required, automation of the workflow as much as possible while retaining flexibility, and user friendliness of operation. Our quantification framework and software fit exactly in current thinking that places emphasis on getting every step of a real-time PCR assay right (such as RNA quality assessment, appropriate reverse transcription, selection of a proper normalization strategy, and so on [2]), especially if small differences between samples need to be reliably demonstrated. In this entire workflow, data analysis is an important last step.

## Results and discussion

### Determination of the error on estimated amplification efficiencies

qBase employs a proven, advanced and universally applicable relative quantification model. An important underlying assumption is that PCR efficiency is assay dependent and sample independent. While this may not be true in every experimental situation, there is currently no consensus on how sample specific PCR efficiencies should be calculated and used for robust quantification. Most evaluation studies attribute a lack of precision to these sample specific efficiency estimation methods. Hence, the gold standard is still the use of a PCR efficiency estimated by a serial dilution series (preferably of pooled cDNA samples, to mimic as much as possible the actual samples to be measured), at least if one aims at accurate and precise quantification. Sample specific PCR efficiency estimation has its usefulness, but currently only for outlier detection [3-5].

Calculation of relative quantities from quantification cycle values requires knowledge of the amplification efficiency of the PCR. As stated above, amplicon specific amplification efficiencies are preferably determined using linear regression (formulas 1 and 5 in Materials and methods) of a serial dilution series with known quantities (either relative or absolute). However, the error on the estimated amplification efficiency is almost never determined, nor taken into account. This error can be calculated using linear regression as well (formulas 2 to 4 and 6), and should subsequently be propagated during conversion of the quantification cycle values to the relative quantities. The formula for the error on the slope provides the mathematical basis to learn how more accurate amplification efficiency estimates can be achieved, that is, by expanding the range of the dilution and including more measurement points.

### Calculation of normalized relative quantities and error minimization

Methods for the conversion of quantification cycle values (Cq; see Materials and methods for terminology) into normalized relative quantities (NRQs) were first reported in 2001. The simplest model described by Livak and Schmittgen [6] assumes 100% PCR efficiency (reflected by a value of 2 for the base *E *of the exponential function) and uses a single reference gene for normalization:

*NRQ *= 2^ΔΔ*Ct*^

Pfaffl [7] modified the above model by adjusting for differences in PCR efficiency between the gene of interest (goi) and a reference gene (ref):

NRQ=EgoiΔCt,goiErefΔCt,ref
 MathType@MTEF@5@5@+=feaafiart1ev1aaatCvAUfeBSjuyZL2yd9gzLbvyNv2Caerbhv2BYDwAHbqedmvETj2BSbqee0evGueE0jxyaibaiKI8=vI8tuQ8FMI8Gi=hEeeu0xXdbba9frFj0=OqFfea0dXdd9vqai=hGuQ8kuc9pgc9s8qqaq=dirpe0xb9q8qiLsFr0=vr0=vr0dc8meaabaqaciGacaGaaeqabaqadeqadaaakeaacaWGobGaamOuaiaadgfacqGH9aqpdaWcaaqaaiaadweadaqhaaWcbaGaam4zaiaad+gacaWGPbaabaGaeuiLdqKaam4qaiaadshacaGGSaGaam4zaiaad+gacaWGPbaaaaGcbaGaamyramaaDaaaleaacaWGYbGaamyzaiaadAgaaeaacqqHuoarcaWGdbGaamiDaiaacYcacaWGYbGaamyzaiaadAgaaaaaaaaa@4B9D@

This model constituted an improvement over the classic delta-delta-Ct method, but cannot deal with multiple (f) reference genes, which is required for reliable measurements of subtle expression differences [8]. Therefore, we further extended this model to take into account multiple stably expressed reference genes for improved normalization. Although not yet published, this advanced and generalized model of relative quantification has been applied previously in our nucleic acid quantification studies [8-12].

NRQ=EgoiΔCt,goi∏ofErefoΔCt,refof
 MathType@MTEF@5@5@+=feaafiart1ev1aaatCvAUfeBSjuyZL2yd9gzLbvyNv2Caerbhv2BYDwAHbqedmvETj2BSbqee0evGueE0jxyaibaiKI8=vI8tuQ8FMI8Gi=hEeeu0xXdbba9frFj0=OqFfea0dXdd9vqai=hGuQ8kuc9pgc9s8qqaq=dirpe0xb9q8qiLsFr0=vr0=vr0dc8meaabaqaciGacaGaaeqabaqadeqadaaakeaacaWGobGaamOuaiaadgfacqGH9aqpdaWcaaqaaiaadweadaqhaaWcbaGaam4zaiaad+gacaWGPbaabaGaeuiLdqKaam4qaiaadshacaGGSaGaam4zaiaad+gacaWGPbaaaaGcbaWaaOqaaeaadaqeWbqaaiaadweadaqhaaWcbaGaamOCaiaadwgacaWGMbWaaSbaaWqaaiaad+gaaeqaaaWcbaGaeuiLdqKaam4qaiaadshacaGGSaGaamOCaiaadwgacaWGMbWaaSbaaWqaaiaad+gaaeqaaaaaaSqaaiaad+gaaeaacaWGMbaaniabg+GivdaaleaacaWGMbaaaaaaaaa@5300@

The calculation of relative quantities, normalization and corresponding error propagation is detailed in formulas 7-16.

**Figure 1:**
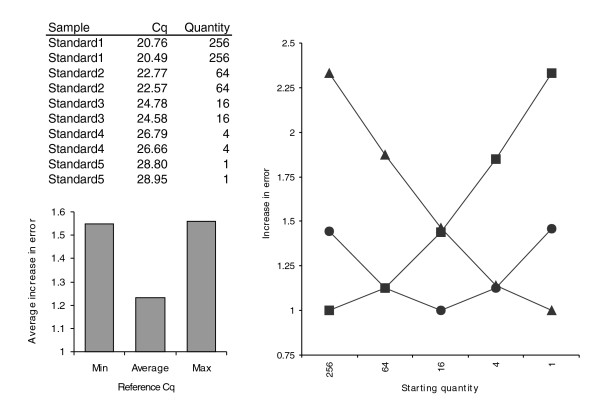
Effect of reference quantification cycle value on increase in error. Relative quantities were calculated for a simulated experiment with a five point four-fold dilution series using, respectively, the lowest Cq (squares), the average Cq (circles) or the highest Cq (triangles) as the reference quantification cycle value. Cq and quantity values are shown at the top left. The increase in the error on relative quantities for the different samples is shown at the top right, with the average increase depicted on the lower left graph.

The basic principle of the delta-Cq quantification model is that a difference (delta) in quantification cycle value between two samples (often a true unknown and calibrator or reference sample) is transformed into relative quantities using the exponential function with the efficiency of the PCR reaction as its base. In principle, any sample can be selected as calibrator, either a real untreated control, or the sample with the highest or lowest expression. In addition, any arbitrary cycle value can be chosen as the calibrator quantification cycle value. The choice of calibrator sample or cycle value does not influence the relative quantification result; while numbers may be different, the actual fold differences between the samples remain identical, so results are fully equivalent and thus only rescaled. However, the choice of calibrator quantification cycle value does have a profound influence on the final error on the relative quantities if the error on the estimated amplification efficiency (see above) is taken into account in the error propagation procedure. To address this issue, we developed an error minimization approach that uses the arithmetic mean quantification cycle value across all samples for a gene within a single run as the calibrator quantification cycle value. As the increase in error is proportional to the difference in quantification cycle between the sample of interest and the calibrator (formula 12), the overall final error is minimized if the mean quantification cycle is used as the calibrator quantification cycle value (Figure 1).

### Evaluation of normalization

The normalization of relative quantities with reference genes relies on the assumption that the reference genes are stably expressed across all tested samples. When using only one reference gene, its stability can not be evaluated. The use of multiple reference genes does not only produce more reliable data, but permits an evaluation of the stability of these genes as well. Previously, we developed a method for the identification of the most stably expressed reference genes in a set of samples [8,13]. The same stability parameter (formulas 21-25) can also be used to evaluate the measured reference genes in an actual quantification experiment. In addition, we calculate here another powerful indicator for expression stability in the actual experiment (formulas 17-20): the coefficient of variation of normalized reference gene relative quantities. Ideally, a reference gene should display the same expression level across all samples after normalization. Consequently, the coefficient of variation indicates how stably the gene is expressed.

To provide reference values for acceptable gene stability values (M) and coefficients of variation (CV), we calculated these normalization quality parameters for our previously established reference gene expression data matrix obtained for 85 samples belonging to 5 different human tissue groups [8]. Table 1 shows that mean CV and M values lower than 25% and 0.5, respectively, are typically observed for stably expressed reference genes in relatively homogeneous sample panels. For more heterogeneous panels, the mean CV and M values can increase to 50% and 1, respectively.

While the use of multiple stably expressed reference genes is currently considered to be the gold standard for normalization of mRNA expression, other strategies might be more appropriate for specific applications, such as: counting cell numbers and expressing mRNA expression levels as copy numbers per cell; using a biologically relevant, specific internal reference (sometimes referred to as *in situ *calibration); or normalizing against DNA (for overview of alternative strategies, see [14]). Clearly, no single strategy is applicable to every experimental situation and it remains up to individual researchers to identify and validate the method most appropriate for their experimental conditions. Important to note is that the presented qBase framework and software is compatible with most of the above mentioned normalization strategies.

### Inter-run calibration

**Figure 2:**
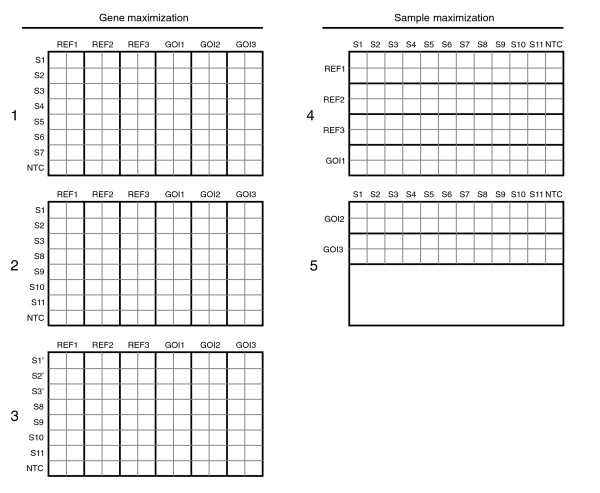
Experimental setup. Experimental setup used to evaluate the effects of inter-run calibration. On the right side, a sample maximization approach is used to analyze 6 genes for 11 samples in 1.5 run. With gene maximization (left side), IRCs (S1, S2, S3) are required to allow comparison of S5-S7 (run 1) to S8-S11 (run 2 or 3), thus requiring two full runs. The IRCs in run 2 are measured on the same cDNA dilution whereas the IRCs in run 3 are measured on newly prepared cDNA from the same RNA.

Two different experimental set-ups can be followed in a qPCR relative quantification experiment. According to the preferred sample maximization method, as many samples as possible are analyzed in the same run. This means that different genes (assays) should be analyzed in different runs if not enough free wells are available to analyze the different genes in the same run. In contrast, the gene maximization set-up analyzes multiple genes in the same run, and spreads samples across runs if required (Figure 2). The latter approach is often used in commercial kits or in prospective studies. It is important to realize that in a relative quantification study, the experimenter is usually interested in comparing the expression level of a particular gene between different samples. Therefore, the sample maximization method is highly recommended because it does not suffer from (often underestimated) technical (run-to-run) variation between the samples.

Whatever set-up is used, inter-run calibration is required to correct for possible run-to-run variation whenever all samples are not analyzed in the same run. For this purpose, the experimenter needs to analyze so-called inter-run calibrators (IRCs); these are identical samples that are tested in both runs. By measuring the difference in quantification cycle or NRQ between the IRCs in both runs, it is possible to calculate a correction or calibration factor to remove the run-to-run difference, and proceed as if all samples were analyzed in the same run.

Inter-run calibration is required because the relationship between quantification cycle value and relative quantity is run dependent due to instrument related variation (PCR block, lamp, filters, detectors, and so on), data analysis settings (baseline correction and threshold), reagents (polymerase, fluorophores, and so on) and optical properties of plastics. Important to note is that inter-run calibration should be performed on a gene per gene basis. It is not sufficient to determine the quantification cycle or relative quantity relation for one primer pair; the experimenter should do this for all assays.

**Figure 3:**
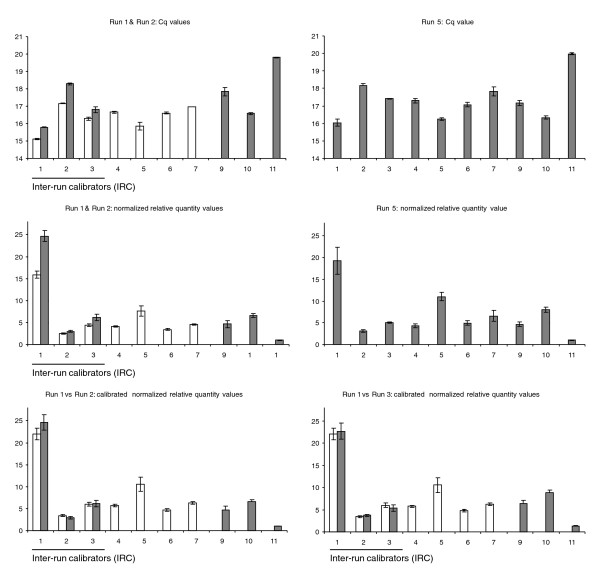
Experimental data comparing sample and gene maximization. The sample maximization approach (run 5) is compared to the gene maximization approach (runs 1 and 2 or 1 and 3). The difference between the IRCs is 0.77 for the Cq values, 72% for the NRQ values, and eliminated after inter-run calibration. Grey and white within the same display item indicates that data comes from different runs.

To provide experimental proof of the advantage of sample maximization over gene maximization with respect to reduction in variation, we designed and performed an experiment consisting of five different runs (Figure 2). The results for one of the genes are shown in Figure 3. With gene maximization, 11 samples are spread over runs 1 and 2. Samples 1 to 3 occur in both runs and can thus be used as IRCs. Run 5 contains all 11 samples in a sample maximization set-up. When comparing the Cq values for the IRCs between runs 1 and 2, it is apparent that those in run 2 are systematically higher (0.77 cycles). After conversion of Cq values into NRQs (and thus taking into account the Cq run-to-run differences for 3 reference genes as well), the NRQ values for samples 1 to 3 differ, on average, by 72% (Additional data file 1). It is important to realize that these values are merely examples. Although the differences can be minimized in a well designed and controlled experiment, they can be much bigger and are generally unpredictable. Anyway, by performing proper inter-run calibration, these run-dependent differences can be corrected and the resulting expression pattern (obtained by calibrating the gene maximization set-up) becomes highly similar to that from the sample maximization method (where there is no run-to-run variation).

To our knowledge, there is only one instrument software that can perform such a correction, but the algorithm is based on the Cq values of a single IRC. Although it can be valid to calibrate data based on Cq values, this method has the drawback that the same template dilution needs to be used in all the runs to be calibrated (for example, nucleic acids from a new cDNA synthesis or a new dilution cannot be reliably used). It is often much more straightforward and easier to calibrate the runs based on the NRQs of the IRCs (formulas 13-16). The quantity (and to some extent also the quality) of the calibrating input material is adjusted after normalization. This has the important advantage that independently prepared cDNA of the same RNA source can be used as a calibrator in the different runs (which allows addition of extra runs, even when the cDNA of the calibrator is run out). To some extent, even a biological replicate (for example, regrown cells) can be used for inter-run calibration when doing the calibration on the NRQs, provided that the experimenter realizes this introduces some level of biological replicate variation (but still adequately removes inter-run variation). The validity of using independently prepared cDNA as calibrator is demonstrated by the experiment described in Figure 2. Inter-run calibration between runs 1 and 3 based on IRCs from different cDNA preparations results in the same expression pattern as that obtained with sample maximization or inter-run calibration with the same cDNA (Figure 3). This is also clearly demonstrated by calculating the ratio of the calibrated NRQs (CNRQs) in runs 2 and 3 (mean ratio: 0.985, 95% CI: [0.945, 1.026]) (Additional data file 2).

It is also advisable to use multiple IRCs. A failed calibrator does not ruin an experiment if two or more are available. In addition, calibration with multiple IRCs gives more precise results with a smaller error. Based on our real calibration experiment, inter-run calibration using a single IRC inherently increases the uncertainty on the relative quantity by about 70% whereas a set of 3 IRCs increases it by only 40% (Table 2). Although it is still advisable to choose the sample maximization setup, inter-run calibration based on the NRQs of multiple IRCs provides reliable results and flexibility in the source of the IRCs.

It is important to note that formulas 13'-16' can only be used for inter-run calibration if the same set of IRCs is used in all runs to be calibrated. For more complex experimental set-ups (whereby different combinations of IRCs are used in the various runs), advanced inter-run calibration algorithms are currently being developed in our laboratory (whereby the challenge is the proper propagation of the errors).

The process of inter-run calibration is very analogous to normalization. Normalization removes the sample specific non-biological variation, while inter-run calibration removes the technical run-to-run variation between samples analyzed in different runs. As such, the same formulas can be used to calculate the inter-run calibration factor (the geometric mean of the different IRCs' NRQs; formulas 13'-16'), and the same quality parameters can be applied to monitor the inter-run calibration process (provided multiple IRCs are used; formulas 21'-25'). Calculation of the IRC stability measure allows the evaluation of the quality of the calibration, which depends on the results of the IRCs. Our experiment shows that, with low M values (Additional data file 2: M ≅ 0.1), virtually identical results are obtained for the different selections of IRCs (Table 2). If inconsistent or erroneous data were obtained for one of the IRCs, higher IRC-M values would be obtained and dissimilar results would be calculated for different sets of IRCs. Therefore, the IRC stability measure M is of great value to determine the quality of the IRCs (provided more than one IRC is used), and to verify whether the calibration procedure is trustworthy.

### qBase

**Figure 4:**
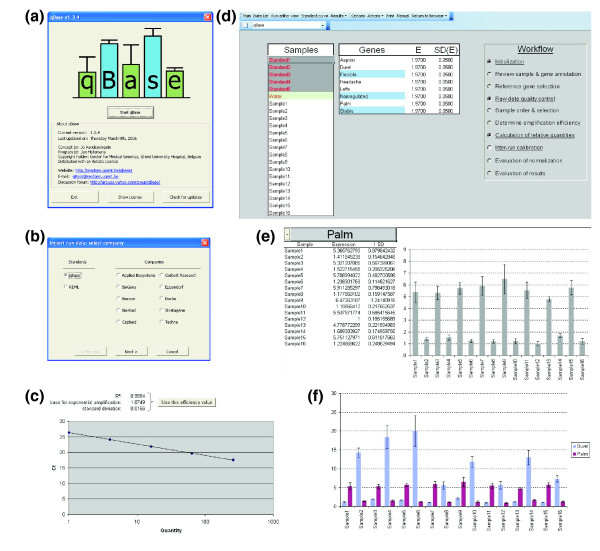
qBase. **(a) **qBase start up screen; **(b) **import wizard allowing selection of the format of the input file; **(c) **standard curve with a five point four-fold dilution series used to calculate the amplification efficiency; **(d) **qBase Analyzer main window with the workflow on the right and sample and gene list on the left - special sample types and reference genes are highlighted; **(e) **single gene histogram; **(f) **multi-gene histogram.

Calculation of NRQs for large data sets, followed by inter-run calibration, is a difficult, error prone and time consuming process when performed in a spreadsheet, especially if errors have to be propagated throughout all calculations. To automate these calculations, and to provide data quality control and result visualization, we developed the software program qBase (Figure 4a). This program is composed of two modules: the 'qBase Browser' for managing and archiving data and the 'qBase Analyzer' for processing raw data into biologically meaningful results.

#### qBase Browser

The Browser allows users to import and to organize hierarchically runs from most currently available qPCR instruments. In qBase, data are structured into three layers: raw data from the individual runs (plates) are stored in the run layer; the experiment layer groups data from different runs that need to be processed and visualized together; and the project layer combines a number of related experiments (for example, biological replicates of the same experiment). This hierarchical structure provides a clear framework to manage qPCR data in a straightforward and simple manner. The qBase Browser window is split into two parts: the bottom of the screen provides an explorer-like window to browse through the data; and the top of the screen contains a separate window displaying the annotation of the selected run, experiment or project. The qBase Browser allows the deletion and addition of projects, experiments and runs. The facility for exporting and importing projects and experiments is a convenient way to exchange data between different qBase users.

#### Data import

Each qPCR instrument has its own method of data collection and storage, accompanied by a large heterogeneity in export files with respect to file format, table layout and used terminology. During import into qBase, the different instrument export files are translated into a common internal format. This format contains information on the well name, sample type, sample and gene name, quantification cycle value, starting quantity values (for standards), and the exclusion status. The last field indicates whether the measurement should be excluded from further calculations without actually discarding the measurement.

Data can be imported from a number of data formats. Two standards (qBase internal format and RDML (Real-time PCR Data Markup Language)) and a number of instrument specific formats are supported. The qBase standard consists of a Microsoft Excel table in which the columns correspond to the information that is used internally by qBase. RDML is a universal format under development for the exchange of qPCR data under the form of XML files [15].

The import wizard guides users through the process of data import (Figure 4b). To address the limitation that some instrument software packages provide only a single identifier field for a well (while there are numerous variables, such as sample and gene name, sample type, and so on), qBase offers the possibility to extract multiple types of information from a single identifier. As such, the identifier 'UNKN|JohnSmith|Gremlin' could, for instance, be extracted to sample type 'UNKN' (unknown), sample name 'JohnSmith' and gene name 'Gremlin'.

#### qBase analyzer

The Analyzer is the data processing module for experiments. It performs relative quantification with proper error propagation along all quantifications, provides a number of quality controls and visualizes NRQs. This process involves several consecutive steps, some of them to be interactively performed by the user, others automatically executed by the program. Users are guided through the analysis by means of a simple workflow scheme in the main screen of the qBase Experiment Analyzer (Figure 4d).

##### Step 1: Initialization

The first step in the workflow is the (automatic) initialization of an experiment, during which raw data from all individual run files from the same experiment are combined into a single data table. The initialization procedure also generates a non-redundant list of all the samples and genes within the experiment. There are no limits on the number of replicates, genes or samples contained within an experiment, except for those imposed by Excel (no more than 65,535 wells can be stored into a single experiment). The absence of such limitations is a major improvement compared to the existing PCR data analysis tools, which are usually limited to processing data from a single plate or run with a fixed number of sample replicates. In qBase, data points with identical sample and gene names are automatically identified as technical replicates, except when the wells are located in different runs. In the latter case, they are interpreted as IRCs and renamed as such, that is, an appendix is added to indicate the run in which they are analyzed. Within the sample and gene lists on the main screen, a color code is used to label the reference genes and special sample types (standards, no template controls, no amplification controls, and IRCs; Figure 4d).

##### Step 2: Review sample and gene annotation

Sample and gene names can be easily modified in all runs belonging to the same experiment. This is very useful for achieving consistent naming of samples and genes across runs. To change names in only a selection of wells in a particular run, a run editor is available in qBase. This editor visualizes the plate (or rotor) layout with well annotation. It allows the modification of gene and sample names, as well as sample types and quantities in individually selected cells or in a range of neighboring cells. Together these tools allow users to review and correct the input annotation.

##### Step 3: Reference gene selection

Accurate relative quantification requires appropriate normalization to correct for non-specific experimental variation, such as differences in starting quantity and quality between the samples. The current consensus is that multiple stably expressed reference genes are required for accurate and robust normalization, especially for measuring subtle expression differences. While different tools are available to determine which candidate reference genes are stably expressed (for example, geNorm [8,13], BestKeeper [16], Normfinder [17]), almost no software is available to perform straightforward normalization using more than one reference gene (with the exception of the commercial Bio-Rad iQ5 and the REST 2005 software). qBase allows gene expression levels to be normalized using up to five reference genes that can easily be selected from the gene list.

##### Step 4: Raw data quality control

Several problems and mistakes can occur when preparing and performing qPCR reactions. The erroneous data produced by these problems need to be detected and excluded from further data analysis to prevent obscuring valuable information or generating false positive results. qBase provides several important quality control checks to evaluate whether: a no template control (NTC) is present for all genes (primer pairs); the quantification cycle values of NTCs are larger than a user defined threshold; the difference in quantification cycle value between samples of interest and NTCs is larger than a user defined threshold; the difference in quantification cycle value between replicated reactions is less than a user defined threshold; and genes are spread over multiple runs (meaning that not all samples tested for a particular gene are analyzed in the same run).

After data quality control, a message box reports all quality issue alerts and the involved data points are color-coded in the data list. This allows users to easily evaluate their data and to select data points for exclusion from analysis without actually removing the data themselves.

##### Step 5: Sample order and selection

During initialization, samples are ordered alphanumerically, but the order of the samples can be adjusted in a user defined way. Samples can be re-ordered in the list by using the up and down keyboard arrows or the sample context menu. Samples that do not need to show up in the results can be excluded by using the delete button on the keyboard or the sample context menu. Apart from changing the default sample order and display selection in the Analyzer main screen, this can also be modified in a temporary gene specific manner when reviewing the results (see below).

##### Step 6: Amplification efficiencies

All quantification models transform (logarithm) quantification cycle values into quantities using an exponential function with the efficiency of the PCR reaction as its base. Although these models and derivative formulas have been used for years, no model or software has taken into account the error (uncertainty) on the calculated efficiency. qBase is the first tool that takes the error on the amplification efficiency into account by means of proper error propagation.

Within qBase, gene specific amplification efficiencies can be specified in three ways. A default amplification efficiency (and error) can be set to all genes, or it can be provided for each gene individually. In the latter case, the efficiencies and corresponding errors can be simply typed (for example, when calculated in an independent experiment), or calculated from a standard dilution series. qBase provides an interface for the evaluation of standard curves whereby outlier reactions can be removed. Amplification efficiencies are calculated by means of linear regression and can be saved to the gene list, in order to be taken into account during further calculation steps (Figure 4c).

##### Step 7: Calculation of relative quantities

**Figure 5:**
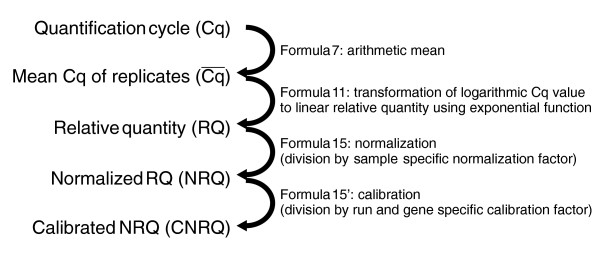
qBase calculation workflow.

After raw qPCR data (quantification cycle values) quality control, reference gene(s) selection and amplification efficiency estimation, qBase can calculate the normalized and rescaled quantities. This process is fully automated and involves the following steps: calculation of the average and the standard deviation of the quantification cycle values for all technical replicates (data points with identical gene and sample names) - the program automatically detects the number of replicates for each sample-gene combination and can deal with a variable number of replicates (formulas 7-8); conversion of quantification cycle values into relative quantities based on the gene specific amplification efficiency (formulas 9-12); calculation of a sample specific normalization factor by taking the geometric mean of the relative quantities of the reference genes (formulas 13-14); normalization of quantities by division by the normalization factor (formulas 15-16); rescaling of the normalized quantities as requested by the user (either relative to the sample with the highest or lowest relative quantity, or relative to a user defined calibrator) (Figure 5). For each step in the calculation of normalized and rescaled relative quantities, qBase propagates the error.

Depending on the settings, qBase will use the classic delta-delta-Ct method (100% PCR efficiency and one reference gene) [6], the Pfaffl modification of delta-delta-Ct (gene specific PCR efficiency correction and one reference gene) [7] or our generalized qBase model (gene specific PCR efficiency correction and multiple reference gene normalization).

##### Evaluation of normalization

Normalization can be monitored by inspecting the normalization factors for all samples, or by calculating reference gene stability parameters. In an experiment with perfect reference genes, identical sample input amounts of equal quality, the normalization factor should be similar for all samples. Variations indicate unequal starting amounts, PCR problems or unstable reference genes. The qBase normalization factor histogram allows easy identification of these potential problems.

One of the unique features of qBase is the option to normalize the relative quantities with multiple reference genes, resulting in more accurate and reliable results. In addition, qBase evaluates the stability of the applied reference genes (and hence the reliability of the normalization) by calculating two quality measures: the coefficient of variation of the normalized reference gene expression levels; and the geNorm stability M-value. Both values are only meaningful, or can be calculated only if multiple reference genes are quantified. The lower these quality values, the more stably the reference genes are expressed in the tested samples. Based on our reported data on the expression of 10 candidate reference genes in 85 samples from 13 different human tissues [8], we have calculated the above mentioned quality parameters and propose acceptable values for M and CV in Table 1. Note that the limits of acceptance largely depend on the required accuracy and resolution of the relative quantification study.

##### Step 8: Inter-run calibration

qBase is especially useful and unique for analysis of experiments containing multiple runs. As users are usually interested in comparing the expression for a given gene between different samples, the sample maximization experimental set-up is the preferred set-up because it minimizes technical (run-to-run) variation between the samples. Nevertheless, the gene maximization set-up is also frequently used. To correct the inter-run variation introduced by this set-up as much as possible, qBase allows runs to be calibrated (on a gene specific basis) using one or multiple IRCs (Figure 5). If no sample(s) is (are) measured for the same gene in the different runs, qBase can not perform calibration and inter-run differences are assumed to be nil. Another unique and important aspect is that inter-run calibration is performed after normalization, which greatly enhances the flexibility in experimental design, as it is no longer obligatory that the same IRC template is used throughout all runs (as such, a new batch of cDNA can be synthesized, and variations will be canceled out during normalization).

##### Step 9: Evaluation of results

Normalized and rescaled relative quantities can be presented in three ways: a single-gene histogram, a multi-gene histogram, or a table. The default sample order and sample selection is defined in the main qBase window by editing the sample list. For the single-gene histogram (Figure 4e) the default order and selection can be changed to an alphanumerical, a user defined or a quantity based (that is, decreasing quantities) order. The option menu allows users to define the size of the error to be displayed (one or more standard error of the mean units). For both histogram views, the scale of the Y-axis can be switched from linear to logarithmic mode and vice versa. The multi-gene histogram (Figure 4f) is instrumental for comparing expression patterns (but not the actual expression levels) between different genes (because each gene is rescaled independently). The genes to be shown in the histogram can be selected from a gene list. Data from the table view (with or without error values) can be easily exported for further processing in other dedicated programs.

#### Distribution

qBase is freely available for non-commercial research and can be downloaded from the qBase website [18].

#### Manual and tutorial

For the training of new qBase users we have designed a demo experiment that is explained in detail in a step-by-step tutorial. Demo experiment 1 consists of 4 runs (96-well format) containing 16 samples, 5 standards, and a no template control to be analyzed for 5 genes of interest and 3 reference genes. Demo experiment 2 adds two runs to the initial experiment, expanding it with eight additional samples and three calibrators for inter-run calibration. After training, complete analysis of these six plates can be performed in less than an hour. This includes data import, correction of well annotation, quality control, determination of amplification efficiencies, inter-run calibration, calculations and results interpretation. To our knowledge, there are no other tools available that can perform all these functions. Conventional spreadsheet calculations would take considerably longer, are error prone and do not include quality control.

## Conclusion

Although qPCR has been around for more than ten years, the employed calculation models are still amenable for improvement. Here we report our advanced, and proven, model for relative quantification that uses gene-specific amplification efficiencies and allows normalization with multiple reference genes. Errors are propagated throughout all calculation steps, and previously ignored errors, such as the uncertainty on the estimated amplification efficiency, are now taken into account. In addition, we developed an inter-run calibration method that allows samples analyzed in different runs to be compared against each other.

We implemented these improved and innovative methods in an easy to use, Microsoft Excel based tool for the management and the automated analysis of qPCR data, coined qBase. This freely available software package incorporates several data quality controls and uses an advanced relative quantification model with efficiency correction, multiple reference gene normalization, inter-run calibration and error propagation along each step of the calculations. A configurable graphical results output and the possibility to import and export experiments allow easy results interpretation and data exchange, respectively.

As a final comment, we would like to point out that, although our framework and program help management and interpretation of mRNA data, assessment of biological relevance or statistical significance requires the correlation of these mRNA data with protein levels or activity, and the measurement of biological replicates, respectively.

## Materials and methods

### Terminology

According to the Real-time PCR Data Markup Language (RDML) we used the proposed universal terms for the plethora of available descriptions (for example, quantification cycle value (Cq) instead of cycle threshold value (Ct), take off point (TOP) or crossing point (Cp)).

### Error propagation

Error propagation is performed using the delta method, based on a truncated Taylor series expansion.

### Symbols used in formulas

N, number of replicates i; g, number of genes j; c, number of IRCs m, m'; r, number of runs l, l'; s, number of samples k; f, number of reference genes p, p'; h, number of standard curve points q with known quantity Q; Cq, quantification cycle; CF, calibration factor; NF, normalization factor; RQ, relative quantity (relative to other samples within the same run for the same gene); NRQ, normalized relative quantity; SE, standard error; IRC, inter-run calibrator; CV, coefficient of variation; A, column matrix in which each element consists of the log_2 _transformed (normalized) relative quantity ratio; V, geNorm pairwise variation; M, geNorm stability parameter.

### Determination of amplification efficiencies

A standard curve can be generated from the Cq and quantity values of a dilution series measured for the same amplicon within a single run. The slope and its standard error can be calculated for this curve by means of linear regression:

slopejl=∑q=1h(Qqjl−Qjl¯)(Cqqjl−Cqjl¯)∑q=1h(Qqjl−Qjl¯)2     (formula 1)
 MathType@MTEF@5@5@+=feaafiart1ev1aaatCvAUfeBSjuyZL2yd9gzLbvyNv2Caerbhv2BYDwAHbqedmvETj2BSbqee0evGueE0jxyaibaiKI8=vI8tuQ8FMI8Gi=hEeeu0xXdbba9frFj0=OqFfea0dXdd9vqai=hGuQ8kuc9pgc9s8qqaq=dirpe0xb9q8qiLsFr0=vr0=vr0dc8meaabaqaciGacaGaaeqabaqadeqadaaakeaacaWGZbGaamiBaiaad+gacaWGWbGaamyzamaaBaaaleaacaWGQbGaamiBaaqabaGccqGH9aqpdaWcaaqaamaaqahabaWaaeWaaeaacaWGrbWaaSbaaSqaaiaadghacaWGQbGaamiBaaqabaGccqGHsisldaqdaaqaaiaadgfadaWgaaWcbaGaamOAaiaadYgaaeqaaaaaaOGaayjkaiaawMcaamaabmaabaGaam4qaiaadghadaWgaaWcbaGaamyCaiaadQgacaWGSbaabeaakiabgkHiTmaanaaabaGaam4qaiaadghadaWgaaWcbaGaamOAaiaadYgaaeqaaaaaaOGaayjkaiaawMcaaaWcbaGaamyCaiabg2da9iaaigdaaeaacaWGObaaniabggHiLdaakeaadaaeWbqaamaabmaabaGaamyuamaaBaaaleaacaWGXbGaamOAaiaadYgaaeqaaOGaeyOeI0Yaa0aaaeaacaWGrbWaaSbaaSqaaiaadQgacaWGSbaabeaaaaaakiaawIcacaGLPaaadaahaaWcbeqaaiaaikdaaaaabaGaamyCaiabg2da9iaaigdaaeaacaWGObaaniabggHiLdaaaOGaaCzcaiaaxMaadaqadaqaaiaabAgacaqGVbGaaeOCaiaab2gacaqG1bGaaeiBaiaabggacaqGGaGaaGymaaGaayjkaiaawMcaaaaa@7058@

se,jl=∑q=1h(Cqqjl,measured−Cqqjl,predicted)2h−2     (formula 2)
 MathType@MTEF@5@5@+=feaafiart1ev1aaatCvAUfeBSjuyZL2yd9gzLbvyNv2Caerbhv2BYDwAHbqedmvETj2BSbqee0evGueE0jxyaibaiKI8=vI8tuQ8FMI8Gi=hEeeu0xXdbba9frFj0=OqFfea0dXdd9vqai=hGuQ8kuc9pgc9s8qqaq=dirpe0xb9q8qiLsFr0=vr0=vr0dc8meaabaqaciGacaGaaeqabaqadeqadaaakeaacaWGZbWaaSbaaSqaaiaadwgacaGGSaGaamOAaiaadYgaaeqaaOGaeyypa0ZaaOaaaeaadaWcaaqaamaaqahabaWaaeWaaeaacaWGdbGaamyCamaaBaaaleaacaWGXbGaamOAaiaadYgacaGGSaGaamyBaiaadwgacaWGHbGaam4CaiaadwhacaWGYbGaamyzaiaadsgaaeqaaOGaeyOeI0Iaam4qaiaadghadaWgaaWcbaGaamyCaiaadQgacaWGSbGaaiilaiaadchacaWGYbGaamyzaiaadsgacaWGPbGaam4yaiaadshacaWGLbGaamizaaqabaaakiaawIcacaGLPaaadaahaaWcbeqaaiaaikdaaaaabaGaamyCaiabg2da9iaaigdaaeaacaWGObaaniabggHiLdaakeaacaWGObGaeyOeI0IaaGOmaaaaaSqabaGccaWLjaGaaCzcamaabmaabaGaaeOzaiaab+gacaqGYbGaaeyBaiaabwhacaqGSbGaaeyyaiaabccacaaIYaaacaGLOaGaayzkaaaaaa@6A74@

sx,jl=1h−1∑q=1h(Qqjl−Qjl¯)2     (formula 3)
 MathType@MTEF@5@5@+=feaafiart1ev1aaatCvAUfeBSjuyZL2yd9gzLbvyNv2Caerbhv2BYDwAHbqedmvETj2BSbqee0evGueE0jxyaibaiKI8=vI8tuQ8FMI8Gi=hEeeu0xXdbba9frFj0=OqFfea0dXdd9vqai=hGuQ8kuc9pgc9s8qqaq=dirpe0xb9q8qiLsFr0=vr0=vr0dc8meaabaqaciGacaGaaeqabaqadeqadaaakeaacaWGZbWaaSbaaSqaaiaadIhacaGGSaGaamOAaiaadYgaaeqaaOGaeyypa0ZaaOaaaeaadaWcaaqaaiaaigdaaeaacaWGObGaeyOeI0IaaGymaaaadaaeWbqaamaabmaabaGaamyuamaaBaaaleaacaWGXbGaamOAaiaadYgaaeqaaOGaeyOeI0Yaa0aaaeaacaWGrbWaaSbaaSqaaiaadQgacaWGSbaabeaaaaaakiaawIcacaGLPaaadaahaaWcbeqaaiaaikdaaaaabaGaamyCaiabg2da9iaaigdaaeaacaWGObaaniabggHiLdaaleqaaOGaaCzcaiaaxMaadaqadaqaaiaabAgacaqGVbGaaeOCaiaab2gacaqG1bGaaeiBaiaabggacaqGGaGaaG4maaGaayjkaiaawMcaaaaa@5744@

SE(slopejl)=se,jlsx,jl(h−1)     (formula 4)
 MathType@MTEF@5@5@+=feaafiart1ev1aaatCvAUfeBSjuyZL2yd9gzLbvyNv2Caerbhv2BYDwAHbqedmvETj2BSbqee0evGueE0jxyaibaiKI8=vI8tuQ8FMI8Gi=hEeeu0xXdbba9frFj0=OqFfea0dXdd9vqai=hGuQ8kuc9pgc9s8qqaq=dirpe0xb9q8qiLsFr0=vr0=vr0dc8meaabaqaciGacaGaaeqabaqadeqadaaakeaacaWGtbGaamyramaabmaabaGaam4CaiaadYgacaWGVbGaamiCaiaadwgadaWgaaWcbaGaamOAaiaadYgaaeqaaaGccaGLOaGaayzkaaGaeyypa0ZaaSaaaeaacaWGZbWaaSbaaSqaaiaadwgacaGGSaGaamOAaiaadYgaaeqaaaGcbaGaam4CamaaBaaaleaacaWG4bGaaiilaiaadQgacaWGSbaabeaakiaacIcacaWGObGaeyOeI0IaaGymaiaacMcaaaGaaCzcaiaaxMaadaqadaqaaiaabAgacaqGVbGaaeOCaiaab2gacaqG1bGaaeiBaiaabggacaqGGaGaaGinaaGaayjkaiaawMcaaaaa@564A@

The base for exponential amplification *E*, and its standard error *SE(E) *are calculated from these values:

Ejl=10(1slopejl)     (formula 5)
 MathType@MTEF@5@5@+=feaafiart1ev1aaatCvAUfeBSjuyZL2yd9gzLbvyNv2Caerbhv2BYDwAHbqedmvETj2BSbqee0evGueE0jxyaibaiKI8=vI8tuQ8FMI8Gi=hEeeu0xXdbba9frFj0=OqFfea0dXdd9vqai=hGuQ8kuc9pgc9s8qqaq=dirpe0xb9q8qiLsFr0=vr0=vr0dc8meaabaqaciGacaGaaeqabaqadeqadaaakeaacaWGfbWaaSbaaSqaaiaadQgacaWGSbaabeaakiabg2da9iaaigdacaaIWaWaaWbaaSqabeaadaqadaqaamaalaaabaGaaGymaaqaaiaadohacaWGSbGaam4BaiaadchacaWGLbWaaSbaaWqaaiaadQgacaWGSbaabeaaaaaaliaawIcacaGLPaaaaaGccaWLjaGaaCzcamaabmaabaGaaeOzaiaab+gacaqGYbGaaeyBaiaabwhacaqGSbGaaeyyaiaabccacaaI1aaacaGLOaGaayzkaaaaaa@4CA1@

SE(Ejl)=Ejl⋅ln⁡(10)⋅SE(slopejl)slopejl  2     (formula 6)
 MathType@MTEF@5@5@+=feaafiart1ev1aaatCvAUfeBSjuyZL2yd9gzLbvyNv2Caerbhv2BYDwAHbqedmvETj2BSbqee0evGueE0jxyaibaiKI8=vI8tuQ8FMI8Gi=hEeeu0xXdbba9frFj0=OqFfea0dXdd9vqai=hGuQ8kuc9pgc9s8qqaq=dirpe0xb9q8qiLsFr0=vr0=vr0dc8meaabaqaciGacaGaaeqabaqadeqadaaakeaacaWGtbGaamyramaabmaabaGaamyramaaBaaaleaacaWGQbGaamiBaaqabaaakiaawIcacaGLPaaacqGH9aqpdaWcaaqaaiaadweadaWgaaWcbaGaamOAaiaadYgaaeqaaOGaeyyXICTaciiBaiaac6gadaqadaqaaiaaigdacaaIWaaacaGLOaGaayzkaaGaeyyXICTaam4uaiaadweadaqadaqaaiaadohacaWGSbGaam4BaiaadchacaWGLbWaaSbaaSqaaiaadQgacaWGSbaabeaaaOGaayjkaiaawMcaaaqaaiaadohacaWGSbGaam4BaiaadchacaWGLbWaa0baaSqaaiaabQgacaqGSbaabaGaaeiiaiaabccacaqGYaaaaaaakiaaxMaacaWLjaWaaeWaaeaacaqGMbGaae4BaiaabkhacaqGTbGaaeyDaiaabYgacaqGHbGaaeiiaiaaiAdaaiaawIcacaGLPaaaaaa@6426@

### Conversion of Cq values into relative quantities

#### Step 1

Calculation of the average Cq value for all replicates of the same gene/sample combination *jk *within a given run *l*:

Cqjkl¯=∑i=1nCqijkln     (formula 7)
 MathType@MTEF@5@5@+=feaafiart1ev1aaatCvAUfeBSjuyZL2yd9gzLbvyNv2Caerbhv2BYDwAHbqedmvETj2BSbqee0evGueE0jxyaibaiKI8=vI8tuQ8FMI8Gi=hEeeu0xXdbba9frFj0=OqFfea0dXdd9vqai=hGuQ8kuc9pgc9s8qqaq=dirpe0xb9q8qiLsFr0=vr0=vr0dc8meaabaqaciGacaGaaeqabaqadeqadaaakeaadaqdaaqaaiaadoeacaWGXbWaaSbaaSqaaiaadQgacaWGRbGaamiBaaqabaaaaOGaeyypa0ZaaSaaaeaadaaeWbqaaiaadoeacaWGXbWaaSbaaSqaaiaadMgacaWGQbGaam4AaiaadYgaaeqaaaqaaiaadMgacqGH9aqpcaaIXaaabaGaamOBaaqdcqGHris5aaGcbaGaamOBaaaacaWLjaGaaCzcamaabmaabaGaaeOzaiaab+gacaqGYbGaaeyBaiaabwhacaqGSbGaaeyyaiaabccacaaI3aaacaGLOaGaayzkaaaaaa@5052@

SE(Cqjkl)=1n(n−1)∑i=1n(Cqijkl−Cqjkl¯)2     (formula 8)
 MathType@MTEF@5@5@+=feaafiart1ev1aaatCvAUfeBSjuyZL2yd9gzLbvyNv2Caerbhv2BYDwAHbqedmvETj2BSbqee0evGueE0jxyaibaiKI8=vI8tuQ8FMI8Gi=hEeeu0xXdbba9frFj0=OqFfea0dXdd9vqai=hGuQ8kuc9pgc9s8qqaq=dirpe0xb9q8qiLsFr0=vr0=vr0dc8meaabaqaciGacaGaaeqabaqadeqadaaakeaacaWGtbGaamyramaabmaabaGaam4qaiaadghadaWgaaWcbaGaamOAaiaadUgacaWGSbaabeaaaOGaayjkaiaawMcaaiabg2da9maakaaabaWaaSaaaeaacaaIXaaabaGaamOBamaabmaabaGaamOBaiabgkHiTiaaigdaaiaawIcacaGLPaaaaaWaaabCaeaadaqadaqaaiaadoeacaWGXbWaaSbaaSqaaiaadMgacaWGQbGaam4AaiaadYgaaeqaaOGaeyOeI0Yaa0aaaeaacaWGdbGaamyCamaaBaaaleaacaWGQbGaam4AaiaadYgaaeqaaaaaaOGaayjkaiaawMcaamaaCaaaleqabaGaaGOmaaaaaeaacaWGPbGaeyypa0JaaGymaaqaaiaad6gaa0GaeyyeIuoaaSqabaGccaWLjaGaaCzcamaabmaabaGaaeOzaiaab+gacaqGYbGaaeyBaiaabwhacaqGSbGaaeyyaiaabccacaaI4aaacaGLOaGaayzkaaaaaa@60A5@

#### Step 2

Transformation of mean Cq value into RQ using the gene specific PCR efficiency *E*_*jl*_, with minimization of the overall error:

Cqrefernce,jl=Cqjl¯=∑k=1sCqjkls     (formula 9)
 MathType@MTEF@5@5@+=feaafiart1ev1aaatCvAUfeBSjuyZL2yd9gzLbvyNv2Caerbhv2BYDwAHbqedmvETj2BSbqee0evGueE0jxyaibaiKI8=vI8tuQ8FMI8Gi=hEeeu0xXdbba9frFj0=OqFfea0dXdd9vqai=hGuQ8kuc9pgc9s8qqaq=dirpe0xb9q8qiLsFr0=vr0=vr0dc8meaabaqaciGacaGaaeqabaqadeqadaaakeaacaWGdbGaamyCamaaBaaaleaacaWGYbGaamyzaiaadAgacaWGLbGaamOCaiaad6gacaWGJbGaamyzaiaacYcacaWGQbGaamiBaaqabaGccqGH9aqpdaqdaaqaaiaadoeacaWGXbWaaSbaaSqaaiaadQgacaWGSbaabeaaaaGccqGH9aqpdaWcaaqaamaaqahabaGaam4qaiaadghadaWgaaWcbaGaamOAaiaadUgacaWGSbaabeaaaeaacaWGRbGaeyypa0JaaGymaaqaaiaadohaa0GaeyyeIuoaaOqaaiaadohaaaGaaCzcaiaaxMaadaqadaqaaiaabAgacaqGVbGaaeOCaiaab2gacaqG1bGaaeiBaiaabggacaqGGaGaaGyoaaGaayjkaiaawMcaaaaa@5B7E@

Δ*Cq*_*jkl *_= *Cq*_*reference*, *jl *_- *Cq*_*jkl *_    (formula 10)

RQjkl=Ejl    ΔCqjkl     (formula 11)
 MathType@MTEF@5@5@+=feaafiart1ev1aaatCvAUfeBSjuyZL2yd9gzLbvyNv2Caerbhv2BYDwAHbqedmvETj2BSbqee0evGueE0jxyaibaiKI8=vI8tuQ8FMI8Gi=hEeeu0xXdbba9frFj0=OqFfea0dXdd9vqai=hGuQ8kuc9pgc9s8qqaq=dirpe0xb9q8qiLsFr0=vr0=vr0dc8meaabaqaciGacaGaaeqabaqadeqadaaakeaacaWGsbGaamyuamaaBaaaleaacaWGQbGaam4AaiaadYgaaeqaaOGaeyypa0JaamyramaaDaaaleaacaWGQbGaamiBaaqaaiaabccacaqGGaGaaeiiaiaabccacqqHuoarcaWGdbGaamyCamaaBaaameaacaWGQbGaam4AaiaadYgaaeqaaaaakiaaxMaacaWLjaWaaeWaaeaacaqGMbGaae4BaiaabkhacaqGTbGaaeyDaiaabYgacaqGHbGaaeiiaiaaigdacaaIXaaacaGLOaGaayzkaaaaaa@4FE5@

SE(RQjkl)=RQjkl2[(ΔCqjkl⋅SD(Ejl)Ejl)2+(ln⁡(Ejl)⋅SD(Cqjkl¯))2]     (formula 12)
 MathType@MTEF@5@5@+=feaafiart1ev1aaatCvAUfeBSjuyZL2yd9gzLbvyNv2Caerbhv2BYDwAHbqedmvETj2BSbqee0evGueE0jxyaibaiKI8=vI8tuQ8FMI8Gi=hEeeu0xXdbba9frFj0=OqFfea0dXdd9vqai=hGuQ8kuc9pgc9s8qqaq=dirpe0xb9q8qiLsFr0=vr0=vr0dc8meaabaqaciGacaGaaeqabaqadeqadaaakeaacaWGtbGaamyramaabmaabaGaamOuaiaadgfadaWgaaWcbaGaamOAaiaadUgacaWGSbaabeaaaOGaayjkaiaawMcaaiabg2da9maakaaabaGaamOuaiaadgfadaqhaaWcbaGaamOAaiaadUgacaWGSbaabaGaaGOmaaaakmaadmaabaWaaeWaaeaafaqabeGabaaabaGaeuiLdqKaam4qaiaadghadaWgaaWcbaGaamOAaiaadUgacaWGSbaabeaakiabgwSixlaadofacaWGebWaaeWaaeaacaWGfbWaaSbaaSqaaiaadQgacaWGSbaabeaaaOGaayjkaiaawMcaaaqaaiaadweadaWgaaWcbaGaamOAaiaadYgaaeqaaaaaaOGaayjkaiaawMcaamaaCaaaleqabaGaaGOmaaaakiabgUcaRmaabmaabaGaciiBaiaac6gadaqadaqaaiaadweadaWgaaWcbaGaamOAaiaadYgaaeqaaaGccaGLOaGaayzkaaGaeyyXICTaam4uaiaadseacaGGOaWaa0aaaeaacaWGdbGaamyCamaaBaaaleaacaWGQbGaam4AaiaadYgaaeqaaaaakiaacMcaaiaawIcacaGLPaaadaahaaWcbeqaaiaaikdaaaaakiaawUfacaGLDbaaaSqabaGccaWLjaGaaCzcamaabmaabaGaaeOzaiaab+gacaqGYbGaaeyBaiaabwhacaqGSbGaaeyyaiaabccacaaIXaGaaGOmaaGaayjkaiaawMcaaaaa@76B5@

### Normalization: inter-run calibration

The procedures for normalization and inter-run calibration are highly analogous and are therefore described in parallel.

#### Step 1

Calculation of the normalization factor *NF *for sample *k *based on the RQs of the reference genes *p*.

#### Step 1'

Calculation of the calibration factor *CF *for gene *j *in run *l *based on the NRQs of the IRCs *m*:

NFk=∏p=1fRQpkf     (formula 13)
 MathType@MTEF@5@5@+=feaafiart1ev1aaatCvAUfeBSjuyZL2yd9gzLbvyNv2Caerbhv2BYDwAHbqedmvETj2BSbqee0evGueE0jxyaibaiKI8=vI8tuQ8FMI8Gi=hEeeu0xXdbba9frFj0=OqFfea0dXdd9vqai=hGuQ8kuc9pgc9s8qqaq=dirpe0xb9q8qiLsFr0=vr0=vr0dc8meaabaqaciGacaGaaeqabaqadeqadaaakeaacaWGobGaamOramaaBaaaleaacaWGRbaabeaakiabg2da9maakeaabaWaaebCaeaacaWGsbGaamyuamaaBaaaleaacaWGWbGaam4AaaqabaaabaGaamiCaiabg2da9iaaigdaaeaacaWGMbaaniabg+GivdaaleaacaWGMbaaaOGaaCzcaiaaxMaadaqadaqaaiaabAgacaqGVbGaaeOCaiaab2gacaqG1bGaaeiBaiaabggacaqGGaGaaGymaiaaiodaaiaawIcacaGLPaaaaaa@4CFF@

CFjl=∏m=1cNRQjlmc
 MathType@MTEF@5@5@+=feaafiart1ev1aaatCvAUfeBSjuyZL2yd9gzLbvyNv2Caerbhv2BYDwAHbqedmvETj2BSbqee0evGueE0jxyaibaiKI8=vI8tuQ8FMI8Gi=hEeeu0xXdbba9frFj0=OqFfea0dXdd9vqai=hGuQ8kuc9pgc9s8qqaq=dirpe0xb9q8qiLsFr0=vr0=vr0dc8meaabaqaciGacaGaaeqabaqadeqadaaakeaacaWGdbGaamOramaaBaaaleaacaWGQbGaamiBaaqabaGccqGH9aqpdaGcbaqaamaarahabaGaamOtaiaadkfacaWGrbWaaSbaaSqaaiaadQgacaWGSbGaamyBaaqabaaabaGaamyBaiabg2da9iaaigdaaeaacaWGJbaaniabg+GivdaaleaacaWGJbaaaaaa@441E@     (formula 13'; for definition of *NRQ*, see formula 15)

SE(NFk)=NFk∑p=1f(SE(RQpk)f⋅RQpk)2     (formula 14)
 MathType@MTEF@5@5@+=feaafiart1ev1aaatCvAUfeBSjuyZL2yd9gzLbvyNv2Caerbhv2BYDwAHbqedmvETj2BSbqee0evGueE0jxyaibaiKI8=vI8tuQ8FMI8Gi=hEeeu0xXdbba9frFj0=OqFfea0dXdd9vqai=hGuQ8kuc9pgc9s8qqaq=dirpe0xb9q8qiLsFr0=vr0=vr0dc8meaabaqaciGacaGaaeqabaqadeqadaaakeaacaWGtbGaamyramaabmaabaGaamOtaiaadAeadaWgaaWcbaGaam4AaaqabaaakiaawIcacaGLPaaacqGH9aqpcaWGobGaamOramaaBaaaleaacaWGRbaabeaakmaakaaabaWaaabCaeaadaqadaqaamaalaaabaGaam4uaiaadweadaqadaqaaiaadkfacaWGrbWaaSbaaSqaaiaadchacaWGRbaabeaaaOGaayjkaiaawMcaaaqaaiaadAgacqGHflY1caWGsbGaamyuamaaBaaaleaacaWGWbGaam4AaaqabaaaaaGccaGLOaGaayzkaaWaaWbaaSqabeaacaaIYaaaaaqaaiaadchacqGH9aqpcaaIXaaabaGaamOzaaqdcqGHris5aaWcbeaakiaaxMaacaWLjaWaaeWaaeaacaqGMbGaae4BaiaabkhacaqGTbGaaeyDaiaabYgacaqGHbGaaeiiaiaaigdacaaI0aaacaGLOaGaayzkaaaaaa@5EC9@

SE(CFjl)=CFjl∑m=1c(SE(NRQjlm)c⋅NRQjlm)2     (formula 14')
 MathType@MTEF@5@5@+=feaafiart1ev1aaatCvAUfeBSjuyZL2yd9gzLbvyNv2Caerbhv2BYDwAHbqedmvETj2BSbqee0evGueE0jxyaibaiKI8=vI8tuQ8FMI8Gi=hEeeu0xXdbba9frFj0=OqFfea0dXdd9vqai=hGuQ8kuc9pgc9s8qqaq=dirpe0xb9q8qiLsFr0=vr0=vr0dc8meaabaqaciGacaGaaeqabaqadeqadaaakeaacaWGtbGaamyramaabmaabaGaam4qaiaadAeadaWgaaWcbaGaamOAaiaadYgaaeqaaaGccaGLOaGaayzkaaGaeyypa0Jaam4qaiaadAeadaWgaaWcbaGaamOAaiaadYgaaeqaaOWaaOaaaeaadaaeWbqaamaabmaabaWaaSaaaeaacaWGtbGaamyramaabmaabaGaamOtaiaadkfacaWGrbWaaSbaaSqaaiaadQgacaWGSbGaamyBaaqabaaakiaawIcacaGLPaaaaeaacaWGJbGaeyyXICTaamOtaiaadkfacaWGrbWaaSbaaSqaaiaadQgacaWGSbGaamyBaaqabaaaaaGccaGLOaGaayzkaaaaleaacaWGTbGaeyypa0JaaGymaaqaaiaadogaa0GaeyyeIuoakmaaCaaaleqabaGaaGOmaaaaaeqaaOGaaCzcaiaaxMaadaqadaqaaiaabAgacaqGVbGaaeOCaiaab2gacaqG1bGaaeiBaiaabggacaqGGaGaaGymaiaaisdacaGGNaaacaGLOaGaayzkaaaaaa@64BF@

#### Step 2

Conversion of RQs into NRQs.

#### Step 2'

Conversion of NRQs into CNRQs:

NRQjk=RQjkNFk     (formula 15)
 MathType@MTEF@5@5@+=feaafiart1ev1aaatCvAUfeBSjuyZL2yd9gzLbvyNv2Caerbhv2BYDwAHbqedmvETj2BSbqee0evGueE0jxyaibaiKI8=vI8tuQ8FMI8Gi=hEeeu0xXdbba9frFj0=OqFfea0dXdd9vqai=hGuQ8kuc9pgc9s8qqaq=dirpe0xb9q8qiLsFr0=vr0=vr0dc8meaabaqaciGacaGaaeqabaqadeqadaaakeaacaWGobGaamOuaiaadgfadaWgaaWcbaGaamOAaiaadUgaaeqaaOGaeyypa0ZaaSaaaeaacaWGsbGaamyuamaaBaaaleaacaWGQbGaam4AaaqabaaakeaacaWGobGaamOramaaBaaaleaacaWGRbaabeaaaaGccaWLjaGaaCzcamaabmaabaGaaeOzaiaab+gacaqGYbGaaeyBaiaabwhacaqGSbGaaeyyaiaabccacaaIXaGaaGynaaGaayjkaiaawMcaaaaa@4AD3@

CNRQjkl=NRQjklCFjl     (formula 15')
 MathType@MTEF@5@5@+=feaafiart1ev1aaatCvAUfeBSjuyZL2yd9gzLbvyNv2Caerbhv2BYDwAHbqedmvETj2BSbqee0evGueE0jxyaibaiKI8=vI8tuQ8FMI8Gi=hEeeu0xXdbba9frFj0=OqFfea0dXdd9vqai=hGuQ8kuc9pgc9s8qqaq=dirpe0xb9q8qiLsFr0=vr0=vr0dc8meaabaqaciGacaGaaeqabaqadeqadaaakeaacaWGdbGaamOtaiaadkfacaWGrbWaaSbaaSqaaiaadQgacaWGRbGaamiBaaqabaGccqGH9aqpdaWcaaqaaiaad6eacaWGsbGaamyuamaaBaaaleaacaWGQbGaam4AaiaadYgaaeqaaaGcbaGaam4qaiaadAeadaWgaaWcbaGaamOAaiaadYgaaeqaaaaakiaaxMaacaWLjaWaaeWaaeaacaqGMbGaae4BaiaabkhacaqGTbGaaeyDaiaabYgacaqGHbGaaeiiaiaaigdacaaI1aGaai4jaaGaayjkaiaawMcaaaaa@4FE0@

SE(NRQjk)=NRQjk(SE(NFk)NFk)2+(SE(RQjk)RQjk)2     (formula 16)
 MathType@MTEF@5@5@+=feaafiart1ev1aaatCvAUfeBSjuyZL2yd9gzLbvyNv2Caerbhv2BYDwAHbqedmvETj2BSbqee0evGueE0jxyaibaiKI8=vI8tuQ8FMI8Gi=hEeeu0xXdbba9frFj0=OqFfea0dXdd9vqai=hGuQ8kuc9pgc9s8qqaq=dirpe0xb9q8qiLsFr0=vr0=vr0dc8meaabaqaciGacaGaaeqabaqadeqadaaakeaacaWGtbGaamyramaabmaabaGaamOtaiaadkfacaWGrbWaaSbaaSqaaiaadQgacaWGRbaabeaaaOGaayjkaiaawMcaaiabg2da9iaad6eacaWGsbGaamyuamaaBaaaleaacaWGQbGaam4AaaqabaGcdaGcaaqaamaabmaabaWaaSaaaeaacaWGtbGaamyramaabmaabaGaamOtaiaadAeadaWgaaWcbaGaam4AaaqabaaakiaawIcacaGLPaaaaeaacaWGobGaamOramaaBaaaleaacaWGRbaabeaaaaaakiaawIcacaGLPaaadaahaaWcbeqaaiaaikdaaaGccqGHRaWkdaqadaqaamaalaaabaGaam4uaiaadweadaqadaqaaiaadkfacaWGrbWaaSbaaSqaaiaadQgacaWGRbaabeaaaOGaayjkaiaawMcaaaqaaiaadkfacaWGrbWaaSbaaSqaaiaadQgacaWGRbaabeaaaaaakiaawIcacaGLPaaadaahaaWcbeqaaiaaikdaaaaabeaakiaaxMaacaWLjaWaaeWaaeaacaqGMbGaae4BaiaabkhacaqGTbGaaeyDaiaabYgacaqGHbGaaeiiaiaaigdacaaI2aaacaGLOaGaayzkaaaaaa@656A@

SE(CNRQjkl)=CNRQjkl(SE(CFjl)CFjl)2+(SE(NRQjkl)NRQjkl)2     (formula 16')
 MathType@MTEF@5@5@+=feaafiart1ev1aaatCvAUfeBSjuyZL2yd9gzLbvyNv2Caerbhv2BYDwAHbqedmvETj2BSbqee0evGueE0jxyaibaiKI8=vI8tuQ8FMI8Gi=hEeeu0xXdbba9frFj0=OqFfea0dXdd9vqai=hGuQ8kuc9pgc9s8qqaq=dirpe0xb9q8qiLsFr0=vr0=vr0dc8meaabaqaciGacaGaaeqabaqadeqadaaakeaacaWGtbGaamyramaabmaabaGaam4qaiaad6eacaWGsbGaamyuamaaBaaaleaacaWGQbGaam4AaiaadYgaaeqaaaGccaGLOaGaayzkaaGaeyypa0Jaam4qaiaad6eacaWGsbGaamyuamaaBaaaleaacaWGQbGaam4AaiaadYgaaeqaaOWaaOaaaeaadaqadaqaamaalaaabaGaam4uaiaadweadaqadaqaaiaadoeacaWGgbWaaSbaaSqaaiaadQgacaWGSbaabeaaaOGaayjkaiaawMcaaaqaaiaadoeacaWGgbWaaSbaaSqaaiaadQgacaWGSbaabeaaaaaakiaawIcacaGLPaaadaahaaWcbeqaaiaaikdaaaGccqGHRaWkdaqadaqaamaalaaabaGaam4uaiaadweadaqadaqaaiaad6eacaWGsbGaamyuamaaBaaaleaacaWGQbGaam4AaiaadYgaaeqaaaGccaGLOaGaayzkaaaabaGaamOtaiaadkfacaWGrbWaaSbaaSqaaiaadQgacaWGRbGaamiBaaqabaaaaaGccaGLOaGaayzkaaWaaWbaaSqabeaacaaIYaaaaaqabaGccaWLjaGaaCzcamaabmaabaGaaeOzaiaab+gacaqGYbGaaeyBaiaabwhacaqGSbGaaeyyaiaabccacaaIXaGaaGOnaiaacEcaaiaawIcacaGLPaaaaaa@6ED9@

### Coefficient of variation of NRQs of a reference gene

#### Step 1

Calculation of the mean NRQ for all samples *k *and a given reference gene *p*:

NRQp¯=∑k=1sNRQpks     (formula 17)
 MathType@MTEF@5@5@+=feaafiart1ev1aaatCvAUfeBSjuyZL2yd9gzLbvyNv2Caerbhv2BYDwAHbqedmvETj2BSbqee0evGueE0jxyaibaiKI8=vI8tuQ8FMI8Gi=hEeeu0xXdbba9frFj0=OqFfea0dXdd9vqai=hGuQ8kuc9pgc9s8qqaq=dirpe0xb9q8qiLsFr0=vr0=vr0dc8meaabaqaciGacaGaaeqabaqadeqadaaakeaadaqdaaqaaiaad6eacaWGsbGaamyuamaaBaaaleaacaWGWbaabeaaaaGccqGH9aqpdaWcaaqaamaaqahabaGaamOtaiaadkfacaWGrbWaaSbaaSqaaiaadchacaWGRbaabeaaaeaacaWGRbGaeyypa0JaaGymaaqaaiaadohaa0GaeyyeIuoaaOqaaiaadohaaaGaaCzcaiaaxMaadaqadaqaaiaabAgacaqGVbGaaeOCaiaab2gacaqG1bGaaeiBaiaabggacaqGGaGaaGymaiaaiEdaaiaawIcacaGLPaaaaaa@4EE9@

SE(NRQp)=1s−1∑k=1s(NRQpk−NRQp¯)2     (formula 18)
 MathType@MTEF@5@5@+=feaafiart1ev1aaatCvAUfeBSjuyZL2yd9gzLbvyNv2Caerbhv2BYDwAHbqedmvETj2BSbqee0evGueE0jxyaibaiKI8=vI8tuQ8FMI8Gi=hEeeu0xXdbba9frFj0=OqFfea0dXdd9vqai=hGuQ8kuc9pgc9s8qqaq=dirpe0xb9q8qiLsFr0=vr0=vr0dc8meaabaqaciGacaGaaeqabaqadeqadaaakeaacaWGtbGaamyramaabmaabaGaamOtaiaadkfacaWGrbWaaSbaaSqaaiaadchaaeqaaaGccaGLOaGaayzkaaGaeyypa0ZaaOaaaeaadaWcaaqaaiaaigdaaeaacaWGZbGaeyOeI0IaaGymaaaadaaeWbqaamaabmaabaGaamOtaiaadkfacaWGrbWaaSbaaSqaaiaadchacaWGRbaabeaakiabgkHiTmaanaaabaGaamOtaiaadkfacaWGrbWaaSbaaSqaaiaadchaaeqaaaaaaOGaayjkaiaawMcaamaaCaaaleqabaGaaGOmaaaaaeaacaWGRbGaeyypa0JaaGymaaqaaiaadohaa0GaeyyeIuoaaSqabaGccaWLjaGaaCzcamaabmaabaGaaeOzaiaab+gacaqGYbGaaeyBaiaabwhacaqGSbGaaeyyaiaabccacaaIXaGaaGioaaGaayjkaiaawMcaaaaa@5BA7@

#### Step 2

Calculation of the coefficient of variation *CV *of a given reference gene *p *across all samples *k*:

CVp=SE(NRQp)NRQp¯     (formula 19)
 MathType@MTEF@5@5@+=feaafiart1ev1aaatCvAUfeBSjuyZL2yd9gzLbvyNv2Caerbhv2BYDwAHbqedmvETj2BSbqee0evGueE0jxyaibaiKI8=vI8tuQ8FMI8Gi=hEeeu0xXdbba9frFj0=OqFfea0dXdd9vqai=hGuQ8kuc9pgc9s8qqaq=dirpe0xb9q8qiLsFr0=vr0=vr0dc8meaabaqaciGacaGaaeqabaqadeqadaaakeaacaWGdbGaamOvamaaBaaaleaacaWGWbaabeaakiabg2da9maalaaabaGaam4uaiaadweadaqadaqaaiaad6eacaWGsbGaamyuamaaBaaaleaacaWGWbaabeaaaOGaayjkaiaawMcaaaqaamaanaaabaGaamOtaiaadkfacaWGrbWaaSbaaSqaaiaadchaaeqaaaaaaaGccaWLjaGaaCzcamaabmaabaGaaeOzaiaab+gacaqGYbGaaeyBaiaabwhacaqGSbGaaeyyaiaabccacaaIXaGaaGyoaaGaayjkaiaawMcaaaaa@4D1C@

#### Step 3

Calculation of the mean coefficient of variation for all reference genes:

CV¯=∑p=1fCVpf     (formula 20)
 MathType@MTEF@5@5@+=feaafiart1ev1aaatCvAUfeBSjuyZL2yd9gzLbvyNv2Caerbhv2BYDwAHbqedmvETj2BSbqee0evGueE0jxyaibaiKI8=vI8tuQ8FMI8Gi=hEeeu0xXdbba9frFj0=OqFfea0dXdd9vqai=hGuQ8kuc9pgc9s8qqaq=dirpe0xb9q8qiLsFr0=vr0=vr0dc8meaabaqaciGacaGaaeqabaqadeqadaaakeaadaqdaaqaaiaadoeacaWGwbaaaiabg2da9maalaaabaWaaabCaeaacaWGdbGaamOvamaaBaaaleaacaWGWbaabeaaaeaacaWGWbGaeyypa0JaaGymaaqaaiaadAgaa0GaeyyeIuoaaOqaaiaadAgaaaGaaCzcaiaaxMaadaqadaqaaiaabAgacaqGVbGaaeOCaiaab2gacaqG1bGaaeiBaiaabggacaqGGaGaaGOmaiaaicdaaiaawIcacaGLPaaaaaa@4AF9@

### Reference gene and IRC stability parameter M

Since normalization and inter-run calibration are highly analogous, quality evaluation using the stability parameter M is similar as well. Therefore, both methods are explained in parallel.

#### Step 1

Calculation of the s × 1 matrix *A*^*gene *^in which the k^th ^element is the log_2 _transformed ratio between the relative quantities (not yet normalized) of two reference genes *p *and *p' *in sample *k*; matrix *A*^*sample *^is calculated in an analogous manner.

#### Step 1'

Calculation of the g × 1 matrix *A*^*irc *^in which the j^th ^element is the log_2 _transformed ratio between the NRQs of two IRCs *m *and *m' *for the same gene *j *within a run *l*; matrix *A*^*run *^is calculated in an analogous manner:

(∀p,p′∈[1,f],p≠p′):App′kgene=log⁡2(RQkpRQkp′)     (formula 21)
 MathType@MTEF@5@5@+=feaafiart1ev1aaatCvAUfeBSjuyZL2yd9gzLbvyNv2Caerbhv2BYDwAHbqedmvETj2BSbqee0evGueE0jxyaibaiKI8=vI8tuQ8FMI8Gi=hEeeu0xXdbba9frFj0=OqFfea0dXdd9vqai=hGuQ8kuc9pgc9s8qqaq=dirpe0xb9q8qiLsFr0=vr0=vr0dc8meaabaqaciGacaGaaeqabaqadeqadaaakeaadaqadaqaaiabgcGiIiaadchacaGGSaGabmiCayaafaGaeyicI48aamWaaeaacaaIXaGaaiilaiaadAgaaiaawUfacaGLDbaacaGGSaGaamiCaiabgcMi5kqadchagaqbaaGaayjkaiaawMcaaiaacQdacaWGbbWaa0baaSqaaiaadchaceWGWbGbauaacaWGRbaabaGaam4zaiaadwgacaWGUbGaamyzaaaakiabg2da9iGacYgacaGGVbGaai4zamaaBaaaleaacaaIYaaabeaakmaabmaabaWaaSaaaeaacaWGsbGaamyuamaaBaaaleaacaWGRbGaamiCaaqabaaakeaacaWGsbGaamyuamaaBaaaleaacaWGRbGabmiCayaafaaabeaaaaaakiaawIcacaGLPaaacaWLjaGaaCzcamaabmaabaGaaeOzaiaab+gacaqGYbGaaeyBaiaabwhacaqGSbGaaeyyaiaabccacaaIYaGaaGymaaGaayjkaiaawMcaaaaa@6428@

(∀m,m′∈[1,c],m≠m′):Amm′jlirc=log⁡2(NRQmjlNRQm′jl)     (formula 21')
 MathType@MTEF@5@5@+=feaafiart1ev1aaatCvAUfeBSjuyZL2yd9gzLbvyNv2Caerbhv2BYDwAHbqedmvETj2BSbqee0evGueE0jxyaibaiKI8=vI8tuQ8FMI8Gi=hEeeu0xXdbba9frFj0=OqFfea0dXdd9vqai=hGuQ8kuc9pgc9s8qqaq=dirpe0xb9q8qiLsFr0=vr0=vr0dc8meaabaqaciGacaGaaeqabaqadeqadaaakeaadaqadaqaaiabgcGiIiaad2gacaGGSaGabmyBayaafaGaeyicI48aamWaaeaacaaIXaGaaiilaiaadogaaiaawUfacaGLDbaacaGGSaGaamyBaiabgcMi5kqad2gagaqbaaGaayjkaiaawMcaaiaacQdacaWGbbWaa0baaSqaaiaad2gaceWGTbGbauaacaWGQbGaamiBaaqaaiaadMgacaWGYbGaam4yaaaakiabg2da9iGacYgacaGGVbGaai4zamaaBaaaleaacaaIYaaabeaakmaabmaabaWaaSaaaeaacaWGobGaamOuaiaadgfadaWgaaWcbaGaamyBaiaadQgacaWGSbaabeaaaOqaaiaad6eacaWGsbGaamyuamaaBaaaleaaceWGTbGbauaacaWGQbGaamiBaaqabaaaaaGccaGLOaGaayzkaaGaaCzcaiaaxMaadaqadaqaaiaabAgacaqGVbGaaeOCaiaab2gacaqG1bGaaeiBaiaabggacaqGGaGaaGOmaiaaigdacaGGNaaacaGLOaGaayzkaaaaaa@6848@

#### Step 2

Calculation of the mean log transformed ratio and the standard deviation *V*^*gene *^for all samples *k *and a given reference gene combination (*p*, *p'*). *V*^*gene *^is the geNorm pairwise variation *V *for two reference genes.

#### Step 2'

Calculation of the mean log transformed ratio and the standard deviation *V*^*irc *^for all runs *l *and a given IRC combination (*m*, *m'*) and a given gene *j*. *V*^*sample *^and *V*^*run *^are calculated similarly from *A*^*sample *^and *A*^*run*^, respectively:

App′gene=∑k=1sApp′kgenes     (formula 22)
 MathType@MTEF@5@5@+=feaafiart1ev1aaatCvAUfeBSjuyZL2yd9gzLbvyNv2Caerbhv2BYDwAHbqedmvETj2BSbqee0evGueE0jxyaibaiKI8=vI8tuQ8FMI8Gi=hEeeu0xXdbba9frFj0=OqFfea0dXdd9vqai=hGuQ8kuc9pgc9s8qqaq=dirpe0xb9q8qiLsFr0=vr0=vr0dc8meaabaqaciGacaGaaeqabaqadeqadaaakeaacaWGbbWaa0baaSqaaiaadchaceWGWbGbauaaaeaacaWGNbGaamyzaiaad6gacaWGLbaaaOGaeyypa0ZaaSaaaeaadaaeWbqaaiaadgeadaqhaaWcbaGaamiCaiqadchagaqbaiaadUgaaeaacaWGNbGaamyzaiaad6gacaWGLbaaaaqaaiaadUgacqGH9aqpcaaIXaaabaGaam4CaaqdcqGHris5aaGcbaGaam4CaaaacaWLjaGaaCzcamaabmaabaGaaeOzaiaab+gacaqGYbGaaeyBaiaabwhacaqGSbGaaeyyaiaabccacaaIYaGaaGOmaaGaayjkaiaawMcaaaaa@54CA@

Amm′jirc=∑l=1rAmm′jlircr     (formula 22')
 MathType@MTEF@5@5@+=feaafiart1ev1aaatCvAUfeBSjuyZL2yd9gzLbvyNv2Caerbhv2BYDwAHbqedmvETj2BSbqee0evGueE0jxyaibaiKI8=vI8tuQ8FMI8Gi=hEeeu0xXdbba9frFj0=OqFfea0dXdd9vqai=hGuQ8kuc9pgc9s8qqaq=dirpe0xb9q8qiLsFr0=vr0=vr0dc8meaabaqaciGacaGaaeqabaqadeqadaaakeaacaWGbbWaa0baaSqaaiaad2gaceWGTbGbauaacaWGQbaabaGaamyAaiaadkhacaWGJbaaaOGaeyypa0ZaaSaaaeaadaaeWbqaaiaadgeadaqhaaWcbaGaamyBaiqad2gagaqbaiaadQgacaWGSbaabaGaamyAaiaadkhacaWGJbaaaaqaaiaadYgacqGH9aqpcaaIXaaabaGaamOCaaqdcqGHris5aaGcbaGaamOCaaaacaWLjaGaaCzcamaabmaabaGaaeOzaiaab+gacaqGYbGaaeyBaiaabwhacaqGSbGaaeyyaiaabccacaaIYaGaaGOmaiaacEcaaiaawIcacaGLPaaaaaa@557B@

Vpp′gene=SD(App′gene)=1s−1∑k=1s(App′kgene−App′gene¯)2     (formula 23)
 MathType@MTEF@5@5@+=feaafiart1ev1aaatCvAUfeBSjuyZL2yd9gzLbvyNv2Caerbhv2BYDwAHbqedmvETj2BSbqee0evGueE0jxyaibaiKI8=vI8tuQ8FMI8Gi=hEeeu0xXdbba9frFj0=OqFfea0dXdd9vqai=hGuQ8kuc9pgc9s8qqaq=dirpe0xb9q8qiLsFr0=vr0=vr0dc8meaabaqaciGacaGaaeqabaqadeqadaaakeaacaWGwbWaa0baaSqaaiaadchaceWGWbGbauaaaeaacaWGNbGaamyzaiaad6gacaWGLbaaaOGaeyypa0Jaam4uaiaadseadaqadaqaaiaadgeadaqhaaWcbaGaamiCaiqadchagaqbaaqaaiaadEgacaWGLbGaamOBaiaadwgaaaaakiaawIcacaGLPaaacqGH9aqpdaGcaaqaamaalaaabaGaaGymaaqaaiaadohacqGHsislcaaIXaaaamaaqahabaWaaeWaaeaacaWGbbWaa0baaSqaaiaadchaceWGWbGbauaacaWGRbaabaGaam4zaiaadwgacaWGUbGaamyzaaaakiabgkHiTmaanaaabaGaamyqamaaDaaaleaacaWGWbGabmiCayaafaaabaGaam4zaiaadwgacaWGUbGaamyzaaaaaaaakiaawIcacaGLPaaadaahaaWcbeqaaiaaikdaaaaabaGaam4Aaiabg2da9iaaigdaaeaacaWGZbaaniabggHiLdaaleqaaOGaaCzcaiaaxMaadaqadaqaaiaabAgacaqGVbGaaeOCaiaab2gacaqG1bGaaeiBaiaabggacaqGGaGaaGOmaiaaiodaaiaawIcacaGLPaaaaaa@6C54@

Vmm′jirc=SD(Amm′jirc)=1r−1∑l=1r(Amm′jlirc−Amm′jirc¯)2     (formula 23')
 MathType@MTEF@5@5@+=feaafiart1ev1aaatCvAUfeBSjuyZL2yd9gzLbvyNv2Caerbhv2BYDwAHbqedmvETj2BSbqee0evGueE0jxyaibaiKI8=vI8tuQ8FMI8Gi=hEeeu0xXdbba9frFj0=OqFfea0dXdd9vqai=hGuQ8kuc9pgc9s8qqaq=dirpe0xb9q8qiLsFr0=vr0=vr0dc8meaabaqaciGacaGaaeqabaqadeqadaaakeaacaWGwbWaa0baaSqaaiaad2gaceWGTbGbauaacaWGQbaabaGaamyAaiaadkhacaWGJbaaaOGaeyypa0Jaam4uaiaadseadaqadaqaaiaadgeadaqhaaWcbaGaamyBaiqad2gagaqbaiaadQgaaeaacaWGPbGaamOCaiaadogaaaaakiaawIcacaGLPaaacqGH9aqpdaGcaaqaamaalaaabaGaaGymaaqaaiaadkhacqGHsislcaaIXaaaamaaqahabaWaaeWaaeaacaWGbbWaa0baaSqaaiaad2gaceWGTbGbauaacaWGQbGaamiBaaqaaiaadMgacaWGYbGaam4yaaaakiabgkHiTmaanaaabaGaamyqamaaDaaaleaacaWGTbGabmyBayaafaGaamOAaaqaaiaadMgacaWGYbGaam4yaaaaaaaakiaawIcacaGLPaaadaahaaWcbeqaaiaaikdaaaaabaGaamiBaiabg2da9iaaigdaaeaacaWGYbaaniabggHiLdaaleqaaOGaaCzcaiaaxMaadaqadaqaaiaabAgacaqGVbGaaeOCaiaab2gacaqG1bGaaeiBaiaabggacaqGGaGaaGOmaiaaiodacaGGNaaacaGLOaGaayzkaaaaaa@6D0B@

#### Step 3

Calculation of the arithmetic mean *M*^*gene *^of all pairwise variations *V*^*gene *^of a given reference gene *p *with all other tested reference genes *p'*. *M*^*gene *^represents the geNorm gene stability measure *M *for a particular reference gene *p*.

#### Step 3'

Calculation of the arithmetic mean *M*^*irc *^of all pair wise variations *V*^*irc *^of a given IRC *m *with all the other IRCs *m'*, for the same gene *j*. *M*^*sample *^and *M*^*run *^are calculated similarly from *V*^*sample *^and *V*^*run*^, respectively:

Mpgene=∑p′=1fVpp′genef−1     (formula 24)
 MathType@MTEF@5@5@+=feaafiart1ev1aaatCvAUfeBSjuyZL2yd9gzLbvyNv2Caerbhv2BYDwAHbqedmvETj2BSbqee0evGueE0jxyaibaiKI8=vI8tuQ8FMI8Gi=hEeeu0xXdbba9frFj0=OqFfea0dXdd9vqai=hGuQ8kuc9pgc9s8qqaq=dirpe0xb9q8qiLsFr0=vr0=vr0dc8meaabaqaciGacaGaaeqabaqadeqadaaakeaacaWGnbWaa0baaSqaaiaadchaaeaacaWGNbGaamyzaiaad6gacaWGLbaaaOGaeyypa0ZaaSaaaeaadaaeWbqaaiaadAfadaqhaaWcbaGaamiCaiqadchagaqbaaqaaiaadEgacaWGLbGaamOBaiaadwgaaaaabaGabmiCayaafaGaeyypa0JaaGymaaqaaiaadAgaa0GaeyyeIuoaaOqaaiaadAgacqGHsislcaaIXaaaaiaaxMaacaWLjaWaaeWaaeaacaqGMbGaae4BaiaabkhacaqGTbGaaeyDaiaabYgacaqGHbGaaeiiaiaaikdacaaI0aaacaGLOaGaayzkaaaaaa@549B@

Mmjirc=∑m′=1cVmm′jircc−1     (formula 24')
 MathType@MTEF@5@5@+=feaafiart1ev1aaatCvAUfeBSjuyZL2yd9gzLbvyNv2Caerbhv2BYDwAHbqedmvETj2BSbqee0evGueE0jxyaibaiKI8=vI8tuQ8FMI8Gi=hEeeu0xXdbba9frFj0=OqFfea0dXdd9vqai=hGuQ8kuc9pgc9s8qqaq=dirpe0xb9q8qiLsFr0=vr0=vr0dc8meaabaqaciGacaGaaeqabaqadeqadaaakeaacaWGnbWaa0baaSqaaiaad2gacaWGQbaabaGaamyAaiaadkhacaWGJbaaaOGaeyypa0ZaaSaaaeaadaaeWbqaaiaadAfadaqhaaWcbaGaamyBaiqad2gagaqbaiaadQgaaeaacaWGPbGaamOCaiaadogaaaaabaGabmyBayaafaGaeyypa0JaaGymaaqaaiaadogaa0GaeyyeIuoaaOqaaiaadogacqGHsislcaaIXaaaaiaaxMaacaWLjaWaaeWaaeaacaqGMbGaae4BaiaabkhacaqGTbGaaeyDaiaabYgacaqGHbGaaeiiaiaaikdacaaI0aGaai4jaaGaayjkaiaawMcaaaaa@5546@

#### Step 4

Calculation of the mean stability measure for all reference genes.

#### Step 4'

Calculation of the mean stability measure for all IRCs:

Mgene¯=∑p=1fMpgenef     (formula 25)
 MathType@MTEF@5@5@+=feaafiart1ev1aaatCvAUfeBSjuyZL2yd9gzLbvyNv2Caerbhv2BYDwAHbqedmvETj2BSbqee0evGueE0jxyaibaiKI8=vI8tuQ8FMI8Gi=hEeeu0xXdbba9frFj0=OqFfea0dXdd9vqai=hGuQ8kuc9pgc9s8qqaq=dirpe0xb9q8qiLsFr0=vr0=vr0dc8meaabaqaciGacaGaaeqabaqadeqadaaakeaadaqdaaqaaiaad2eadaahaaWcbeqaaiaadEgacaWGLbGaamOBaiaadwgaaaaaaOGaeyypa0ZaaSaaaeaadaaeWbqaaiaad2eadaqhaaWcbaGaamiCaaqaaiaadEgacaWGLbGaamOBaiaadwgaaaaabaGaamiCaiabg2da9iaaigdaaeaacaWGMbaaniabggHiLdaakeaacaWGMbaaaiaaxMaacaWLjaWaaeWaaeaacaqGMbGaae4BaiaabkhacaqGTbGaaeyDaiaabYgacaqGHbGaaeiiaiaaikdacaaI1aaacaGLOaGaayzkaaaaaa@50FA@

Mjirc¯=∑m=1fMmjircf     (formula 25')
 MathType@MTEF@5@5@+=feaafiart1ev1aaatCvAUfeBSjuyZL2yd9gzLbvyNv2Caerbhv2BYDwAHbqedmvETj2BSbqee0evGueE0jxyaibaiKI8=vI8tuQ8FMI8Gi=hEeeu0xXdbba9frFj0=OqFfea0dXdd9vqai=hGuQ8kuc9pgc9s8qqaq=dirpe0xb9q8qiLsFr0=vr0=vr0dc8meaabaqaciGacaGaaeqabaqadeqadaaakeaadaqdaaqaaiaad2eadaqhaaWcbaGaamOAaaqaaiaadMgacaWGYbGaam4yaaaaaaGccqGH9aqpdaWcaaqaamaaqahabaGaamytamaaDaaaleaacaWGTbGaamOAaaqaaiaadMgacaWGYbGaam4yaaaaaeaacaWGTbGaeyypa0JaaGymaaqaaiaadAgaa0GaeyyeIuoaaOqaaiaadAgaaaGaaCzcaiaaxMaadaqadaqaaiaabAgacaqGVbGaaeOCaiaab2gacaqG1bGaaeiBaiaabggacaqGGaGaaGOmaiaaiwdacaGGNaaacaGLOaGaayzkaaaaaa@51B1@

### Calculations on the effect of inter-run calibration

The calculations for Figure 3 and Additional data file 1 have been performed as described in the formulas listed above. Difference in Cq is defined as the mean difference between the IRCs in run 1 and run 2. Fold change is defined as the ratio of the geometric mean of the (C)NRQs of the IRCs in run 1 and run 2.

For the calculation of the effects of inter-run calibration, NRQ values were retrieved from qBase for runs 1, 2 and 3 independently. Inter-run calibration was performed as described in formulas 13'-16', using one, two or three IRCs (Additional data file 2). The effect of inter-run calibration with two IRCs was calculated on the three sets of two IRCs (IRCs 1,2 versus IRCs 1,3 versus IRCs 2,3). Similarly, the effect of inter-run calibration with one IRC was calculated over all individual IRCs.

The increase in error is defined as the ratio of the relative error after and before calibration. The 95% confidence interval (CI) for this increase was calculated on log-transformed ratios. For the investigation of the effect of the selection of (sets of) IRCs from the three available calibrators, CNRQs for the different calibrated data sets were rescaled to allow them to be compared. The fold difference between the data sets was log transformed and a 95% CI was calculated. The effect of calibration with identical or independently prepared cDNA was studied similarly to the effect of the selection of IRCs. The IRC stability measure was calculated as described in formulas 21'-25'.

## Additional data files

The following additional data are available with the online version of this paper. Additional data file 1 contains all the data and calculations leading to the results presented in Figure 3. Additional data file 2 contains all the data and calculations that were used for the evaluation of the effect of inter-run calibration on the final results. The conclusions of these calculations are represented, in part, in Table 2.

## Supplementary Material

Additional data file 1 Data and calculations leading to the results presented in Figure 3 Click here for file

Additional data file 2 The conclusions of these calculations are represented, in part, in Table 2 Click here for file

## Figures and Tables

**Table 1:** Reference gene expression stability evaluation

Tissue type	Gene	CV (%)	M	Mean CV (%)	Mean M
Neuroblastoma	*UBC*	31.84	0.740	30.89	0.703
	*SDHA*	27.40	0.660		
	*HPRT1*	37.11	0.736		
	*GAPDH*	27.21	0.675		
Fibroblast	*YHWAZ*	18.19	0.408	14.81	0.365
	*HPRT1*	8.84	0.308		
	*GAPDH*	17.40	0.378		
Leukocyte	*B2M*	15.76	0.400	15.81	0.394
	*UBC*	15.79	0.389		
	*YWHAZ*	15.89	0.393		
Bone marrow	*YWHAZ*	17.77	0.383	15.47	0.372
	*UBC*	13.60	0.356		
	*RPL13A*	15.03	0.376		
Normal pool	*TBP*	47.51	1.099	43.73	0.925
	*HPRT1*	46.99	0.988		
	*HMBS*	31.16	0.849		
	*SDHA*	49.50	0.869		
	*GAPDH*	43.50	0.819		

**Table 2:** Effects of the number and selection of IRCs on the increase in error and the fold difference between calibrated NRQs

	Increase in error		Fold difference between calibrated normalized quantities	
		
	Mean [95% CI]	Max	Mean [95% CI]	Max
1 IRC				
run1-run2	1.684 [1.579,1.797]	10.98	1.048 [1.034,1.061]	1.143
run1-run3	1.68 [1.576,1.79]	10.98	1.053 [1.038,1.067]	1.135
2 IRCs				
run1-run2	1.374 [1.289,1.466]	7.73	1.024 [1.017,1.03]	1.069
run1-run3	1.489 [1.415,1.567]	7.73	1.026 [1.019,1.033]	1.065
3 IRCs				
run1-run2	1.399 [1.292,1.513]	5.28		
run1-run3	1.394 [1.288,1.508]	5.28		
